# Liquid Biopsies in Sarcoma Clinical Practice: Where Do We Stand?

**DOI:** 10.3390/biomedicines9101315

**Published:** 2021-09-26

**Authors:** Pia van der Laan, Winan J. van Houdt, Daan van den Broek, Neeltje Steeghs, Winette T. A. van der Graaf

**Affiliations:** 1 Department of Surgical Oncology, Netherlands Cancer Institute, 1066 CX Amsterdam, The Netherlands; p.vd.laan@nki.nl (P.v.d.L.); w.v.houdt@nki.nl (W.J.v.H.); 2 Department of Medical Oncology, Netherlands Cancer Institute, 1066 CX Amsterdam, The Netherlands; n.steeghs@nki.nl; 3 Department of Laboratory Medicine, Netherlands Cancer Institute, 1066 CX Amsterdam, The Netherlands; da.vd.broek@nki.nl; 4 Department of Medical Oncology, Erasmus MC Cancer Institute, Erasmus MC, 3015 GD Rotterdam, The Netherlands

**Keywords:** sarcoma, liquid biopsy, biomarker, CTC, ctDNA, cell-free DNA, clinical practice

## Abstract

Sarcomas are rare tumors of bone and soft tissue with a mesenchymal origin. This uncommon type of cancer is marked by a high heterogeneity, consisting of over 70 subtypes. Because of this broad spectrum, their treatment requires a subtype-specific therapeutic approach. Tissue biopsy is currently the golden standard for sarcoma diagnosis, but it has its limitations. Over the recent years, methods to detect, characterize, and monitor cancer through liquid biopsy have evolved rapidly. The analysis of circulating biomarkers in peripheral blood, such as circulating tumor cells (CTC) or circulating tumor DNA (ctDNA), could provide real-time information on tumor genetics, disease state, and resistance mechanisms. Furthermore, it traces tumor evolution and can assess tumor heterogeneity. Although the first results in sarcomas are encouraging, there are technical challenges that need to be addressed for implementation in clinical practice. Here, we summarize current knowledge about liquid biopsies in sarcomas and elaborate on different strategies to integrate liquid biopsy into sarcoma clinical care.

## 1. Introduction

During the past decades, non-invasive methods to detect and monitor cancer have gained a lot of attention. Liquid biopsy is a technique to detect biomarkers circulating in body fluids, primarily blood. Biomarkers detected by liquid biopsy include circulating tumor cells or nucleic acids, exosomes, tumor educated platelets, and others, providing information on the feature of primary tumors or metastases [1,2]. The detection of circulating tumor cells (CTCs) and circulating tumor DNA (ctDNA) as biomarkers are particularly well developed. CTCs are shed by the primary tumor and can be captured while circulating through the bloodstream, and might reflect early metastatic spread. CTCs can be enriched from the peripheral blood, using physical (size and density) features or biological (cell surface markers and specific mutations or translocations) properties and analyzed for proteins, RNA, and DNA [3,4]. More recently, ctDNA has been subject to many studies as another attractive biomarker. ctDNA, as a part of the total cell-free DNA (cfDNA) present in the circulation, is released into the patients’ blood by dying or degrading cancer cells, and may reflect genetic aberrations of the tumor genome. The presence of ctDNA in the plasma is associated with genotype, tumor burden, and necrosis [5,6,7]. Tumor derived DNA can be analyzed for quantity and integrity, but also for DNA profiling or mutation detection. In addition to CTCs and ctDNA, other types of circulating material such as RNA or tumor-educated platelets (TEP) can also be analyzed from the systemic circulation [8,9].

Although tissue biopsy is the current golden standard for cancer diagnosis and evaluation, it has some limitations. First, tissue biopsy is an invasive procedure, and has a risk of complications, which makes repeated sampling unattractive. Second, tissue biopsy is not always feasible due to the location of the tumor. Third, a single tissue biopsy may not represent tumor heterogeneity and, lastly, it will not track genetic changes during the disease course [1,2]. Liquid biopsy is an appealing alternative, aimed to derive information similar to what is normally obtained from tissue biopsy. This approach may provide a less invasive and easier obtainable alternative to tissue-based methods. An increasing body of evidence has demonstrated clinical utility for the use of liquid biopsies in various solid malignancies. As an example, it was observed in melanoma that ctDNA samples can provide *BRAF* and *NRAS* genotypes as surrogates for tissue diagnostics, with a high degree of concordance compared with tissue testing [10,11]. In addition, for non-small-cell lung cancer and breast cancer, the use of liquid biopsy to detect somatic alterations, to predict recurrence, and to monitor treatment response has extensively been studied [12,13,14]. Therefore, blood-based analyses may be useful for many purposes, enabled by repeated and longitudinal sampling [15,16].

Unfortunately, for rare cancer types such as sarcoma, the number of studies evaluating liquid biopsies are relatively low. Sarcomas form a heterogeneous group of malignant tumors arising in bone and soft tissues throughout the body, originating from mesenchymal cells. This uncommon group of tumors accounts for 1% of adult malignancies and consists of over 70 subtypes according to the WHO [17]. From a genetical point of view, sarcomas can be classified into two broad categories: sarcomas with a simple karyotype characterized by a translocation or a specific mutation, and sarcomas with a more complex karyotype containing multiple gains, losses, and amplifications [18,19]. As every single subtype has distinctive characteristics and behavior, treatment of sarcoma requires a subtype-specific approach. Surgical resection is the mainstay of treatment, often with curative intent, whereas for well-defined soft tissue and Ewing sarcomas, (neo)adjuvant radiotherapy is given and, on indication, systemic treatment is part of the primary treatment. For patients with advanced or irresectable disease, the prognosis is generally poor, and palliative systemic chemotherapy and local radiotherapy are meant to relieve symptoms and/or prolong life.

Because of the rarity and large heterogeneity of sarcomas, studies on liquid biopsy in this cancer type are usually limited to a small number of patients per subtype, which makes it difficult to demonstrate its prognostic value and clinical utility. This review aims to describe the potential applications of liquid biopsy in sarcomas for both simple karyotype and complex karyotype sarcomas, which require different strategies. In addition, we will summarize recent advantages, challenges, and perspectives in the area of liquid biopsy in sarcomas.

## 2. Liquid Biopsy in Sarcoma Clinical Practice

The detection of biomarkers used for liquid biopsy in sarcomas is challenging due to their low concentrations. Current detection methods are mainly PCR or sequencing based. For ctDNA analysis, digital droplet PCR (ddPCR) and next generation sequencing (NGS) are the most frequently used methods. A comparison between these techniques and their strengths and weaknesses is described in Table 1.

We will discuss relevant studies with potential clinical applications of liquid biopsies first. Next, we will describe studies with different assays used in various soft tissue sarcomas (STS) at different stages of the disease, which are summarized in Table 2 and Table 3.

### 2.1. Diagnosis

Blood-based diagnostics might provide an alternative option for a histological biopsy if no lesions are accessible for biopsy, or if a tissue biopsy is deemed to have a risk for complications. Gastro-intestinal stromal tumors (GISTs), accounting for around 20% of STS [24], are one of the most studied sarcomas for ctDNA analysis. Most GISTs harbor either an oncogenic activating *KIT* or *PDGFRA* mutation [25,26]; around two-thirds of these *KIT* mutations are in exon 11, and less common mutations are in exons 9, 13, 17, or 8 [27]. Boonstra et al. described a ddPCR assay to specifically detect exon 11 *KIT* mutations in the ctDNA of GIST patients with known *KIT* exon 11 mutations, and were able to detect this mutation with a sensitivity of 95%, suggesting liquid biopsy as an alternative source for tissue biopsy [28]. The same investigators subsequently published a case report in which they used ctDNA to analyze the presence of mutations in a patient for whom tissue biopsy was not feasible because of a high risk of bleeding. Extracting cfDNA from this patient’s plasma followed by profiling by NGS revealed a mutation in *PDGFRA*, confirming a GIST tumor. The number of mutated DNA copies decreased after starting treatment with the tyrosine kinase inhibitor (TKI) imatinib, which was in agreement with the observed response on the imaging [29].

In particular, GISTs are well suited for liquid biopsy because of their characteristic mutations, whereas this will be more challenging for sarcomas with a more complex karyotype. Recently, Szymanski et al. demonstrated the NGS of plasma ctDNA to distinguish malignant peripheral nerve sheath tumor (MPNST) from benign plexiform neurofibroma (PN). MPNST may arise from PN, but screening for this transformation remains challenging due to different aspects, such as the heterogeneity of the lesions, complicating radiographic diagnosis and the accuracy of the tissue biopsy. In this study, a total of 107 plasma samples of 73 MPNST patients, PN patients, or healthy individuals were analyzed for copy number alterations (CNA) to estimate the total amount of cfDNA derived from the tumor (tumor fraction). It was shown that profiling plasma cfDNA can reliably distinguish malignant tumors from their pre-malignant counterparts using tumor fraction, with a sensitivity of 58% and a specificity of 91%. The tumor fraction in the plasma and the cfDNA fragment length showed significant differences between healthy controls, PN patients, and MPNST patients. By correlating serial plasma samples of MPNST patients to disease burden on imaging, the sum of the longest tumor diameters on the imaging were correlated significantly with the tumor fractions in the plasma. The authors suggest this method for early cancer detection and monitoring of cancer-predisposed populations such as neurofibromatosis [30]. Yokoi et al. tested a liquid biopsy approach using circulating micro RNA (miRNA) to help gynecologists preoperatively differentiate between a benign leiomyoma or a malignant leiomyosarcoma (LMS), which is challenging as they appear to be similar on imaging and the sensitivity of preoperative endometrial sampling is low. Accurate pre-operative diagnosis is crucial for selecting cases suitable for laparoscopic surgery, as this type of surgery is often done with tumor morcellation (intra-operative fragmentation), which in case of a malignancy can spread tumor cells throughout the peritoneal cavity. In this study, miRNA expression profiles were analyzed for a total of 90 serum samples to distinguish benign from malignant tumors. miRNA profiling showed a distinct pattern of uterine LMS compared with benign tumors, and a total of seven miRNAs were identified as potential biomarker candidates. Although the results of this study need to be validated in a larger cohort, this study shows serum miRNA profiling as a biomarker for the selection of cases potentially eligible for laparoscopic surgery with morcellation [31].

### 2.2. Follow-Up

Follow-up protocols in sarcoma care generally consist of physical examination and serial imaging of any kind. While frequent imaging with CT scans is inconvenient and has the disadvantage of—albeit low—radiation exposure, liquid biopsy enables the option of low-risk, easy repeated sampling during routine blood draws. However, low levels of circulating material in early stage cancer pose a challenge for using liquid biopsy as marker of early disease recurrence [2]. A study by Eastley et al. aimed to study the levels of total cfDNA in blood samples to monitor change in disease during the follow-up of multiple sarcoma subtypes. Matched intra- and post-operative samples of non-metastatic patients were available for 22 patients; no significant drop in total cfDNA levels after surgery was found. In addition, total cfDNA levels post-operatively were compared with matched levels at the point of disease recurrence and were not shown to be significantly different [32]. This is in contrast with an earlier study by the same authors, in which significantly elevated cfDNA levels were found in the samples of metastatic sarcoma patients of different subtypes, positively correlated with disease burden [33]. This suggests more potential of cfDNA as a biomarker for sarcomas within the metastatic setting than during follow up after curative treatment. Additional larger, prospective studies are necessary to draw any firm conclusions on the potential of liquid biopsy-based in patients on surveillance.

### 2.3. Monitoring and Treatment Selection

Despite advances in identifying molecular targets for therapy, chemotherapy remains the standard of care for inoperable, advanced, and metastatic sarcomas. It is well-known that conventional chemotherapeutic agents are associated with many side effects and can result in long-term toxicity. A biomarker capable of predicting chemotherapy response more accurately could prevent patients from unnecessary treatment with these toxic therapies. Only a few studies have investigated liquid biopsy for the purpose of predicting response to chemotherapy. The Ewing specific fusion product was studied by Krumbholz et al., applying ddPCR in 234 blood samples from 20 patients at the start and during the treatment of Ewing sarcoma so as to predict the chemotherapy response. Patient-specific primer sets were used for the detection of the fusion sequence by PCR at initial diagnosis and relapse. Fusion sequence ctDNA copy numbers were detected in 18/20 plasma samples and the number of copies showed a correlation with tumor volume. In addition, follow-up samples were collected in 17 patients to evaluate the genomic fusion sequence as a marker for therapy response. In two patients, no detectable ctDNA copies were detected in any of the follow-up samples. A fast reduction of ctDNA copy numbers was observed in the majority of patients: 9/15 of patients had no fusion sequence detectable at the start of the second cycle of chemotherapy. Of the remaining six patients, three were negative at start of the third cycle. Three patients relapsed during the study, all indicated by an increase in ctDNA copy numbers of the fusion sequence [34].

A clinical study by Martín-Broto et al. evaluated the feasibility of using CTCs as a liquid biomarker in metastatic soft tissue sarcoma treated with olaratumab monotherapy for one cycle, followed by olaratumab plus doxorubicin for up to six cycles. Blood samples of 35 patients were available for CTC determination and collected during the first three cycles of therapy. Decrease in CTC numbers after olaratumab monotherapy was seen in 11/19 patients (57.9%) with disease control (response or stable disease) and 5/16 patients (31.2%) without disease control. In several patients, an increase in CTCs during the first cycle was observed, followed by a decrease in CTCs by cycle two. However, the results did not reach statistical significance, probably due to the small study size [35].

An increasing number of studies are using ctDNA mutation analysis to select patients who will benefit from targeted therapy. For various cancer types, circulating tumor DNA profiling has been assessed for select cases who will benefit from therapy and to detect primary resistance to these therapies [36,37,38]. The cobas^®^ EGFR Mutation Test v2 was the first PCR-based assay approved by the U.S. Food and Drug Administration (FDA) using circulating cfDNA for the detection of mutations in the epidermal growth factor receptor (*EGFR*) gene to identify patients with metastatic NSCLC eligible for treatment with the TKI erlotinib [2,39]. More recently, the FDA approved two liquid biopsy tests, Guardant360^®^ CDx and FoundationOne^®^ Liquid CDx, which check for multiple genetic changes to match this with the best treatment option in solid malignancies [40,41]. ctDNA analysis using Guardant360^®^ was explored for both GIST and LMS. In a study of 73 LMS patients, 59 patients were found to have an alteration detected by the NGS panel. The most common alterations found by this panel were in *TP53*, *BRAF*, *CCNE*, *EGFR*, *PIK3CA*, *FGFR1*, *RB1*, *KIT*, and *PDGFRA* [42]. Unfortunately, most drugs targeting these alterations have not shown to be successful for the treatment of LMS until now. In a study in 243 GIST patients in different disease stages, the NGS panel detected mutations in 45% of patients. None of the patients with localized GIST had detectable DNA, however, in metastatic patients, this NGS panel was able to identify a driver mutation, thereby guiding the optimal therapy [43]. A similar approach of liquid biopsy to guide therapy selection using ddPCR was explored in GIST patients using liquid biopsy to detect mutations, which can be targeted by the TKI imatinib [28]. Before the start of imatinib treatment, a mutation-specific ddPCR assay was designed to assess the exact mutation status in plasma samples derived from 22 patients. Mutations in ctDNA were detected in 13 of 14 metastasized patients, whereas the detection rate in localized disease was only found in one out of eight patients. By mutation analysis of ctDNA, other researchers identified *TP53* mutations in wild-type GISTs—usually resistant to imatinib—and found increased allele frequency of this mutation during progression, suggesting a rapid clonal selection during tumor progression while on imatinib treatment [44]. Different groups also found evidence for the appearance of secondary mutations in GIST after imatinib treatment [45,46]. For liposarcoma, its was shown by Jung et al. that *TP53* mutant clones found in circulating cfDNA emerge during HDM2 inhibitor treatment of de-differentiated liposarcoma [47]. These longitudinal mutation analyses suggest liquid biopsy as a tool to indicate early therapy resistance and could thereby prevent unnecessary treatment.

Another scenario to use liquid biopsy to monitor treatment is to differentiate response to systemic therapy from progression in neo-adjuvant therapy, which can be quite challenging for mesenchymal tumors. The literature shows that the assessment of tumor response of sarcomas treated with chemotherapy and radiation based on imaging only may not be sufficient to represent the actual tumor activity and thus response [48]. It has been shown for GIST that, particularly during treatment with TKI, using the RECIST measurement often underestimates the therapeutic effect [49,50]. The application of liquid biopsy to differentiate pseudoprogression from actual progression using a longitudinal ctDNA profile combined with radiological findings could potentially overcome these problems.

## 3. Liquid Biopsy for Simple Karyotype Sarcomas

### 3.1. Synovial Sarcoma

Synovial sarcoma is characterized by the chromosomal translocation t(X;18)(p11.2;q11.2), resulting in the fusion of two genes: the *SYT* (or *SS18*) gene on chromosome 18 to either *SSX1* or *SSX2*, or *SSX3* on chromosome X. This fusion occurs independently of histological subtype, which can either be biphasic or monophasic [71]. Since *SYT-SSX* is present in up to 90% of synovial sarcomas [72], this specific alteration may provide a tool for diagnostics and monitoring. Several studies have investigated the potential for detecting this fusion product in peripheral blood samples. Hashimoto et al. described a case report for which peripheral blood samples were collected to perform PCR on circulating tumor cells. Blood was collected at primary diagnosis, after resection, and after the first cycle of chemotherapy. In this patient, the *SYT-SSX* fusion was detected at primary diagnosis, whereas the fusion gene was not detectable after resection and after first chemotherapy, even though multiple lung metastases had developed [73]. Mihály et al. collected blood samples for the CTC analysis of 15 synovial sarcoma patients every six months after treatment by surgery, systemic therapy, or radiotherapy. Samples were obtained from patients in various disease stages, of which the majority had recurrent or metastatic disease (12 out of 15). The RNA was isolated, and nested PCR and ddPCR, two methods to improve sensitivity of conventional PCR, were performed. Fusion transcript was identified by ddPCR in only one case. Nested PCR could not detect the fusion product in any of the cases. They concluded that the detection of a fusion gene after treatment is difficult, and therefore insufficient for monitoring tumor recurrence [51]. These results are supported by a study of Przybyl et al., where RNA was isolated from 38 blood samples of synovial sarcoma patients to perform nested RT-PCR on CTCs. This resulted in a detection in 2 out of 38 samples, both patients with localized disease at the time of blood collection. They concluded that this CTC approach is not sensitive enough in patients with synovial sarcoma and suggested ctDNA to be more clinically useful for prognostication, molecular profiling, and surveillance [52]. Ogino et al. studied a cfDNA-based approach in a case report of a young woman with gastric synovial sarcoma. Blood samples were collected before surgical resection, one month after resection, and six months after resection. Quantitative PCR (qPCR) was performed on the ctDNA and showed the fusion sequence in the preoperative sample, while it was not detected in the postoperative samples [74]. Other circulating markers have also been explored as potential biomarkers in synovial sarcoma. The miRNA profiling of nine synovial sarcoma patients showed the serum miR-92b-3p to be upregulated in synovial sarcoma patients. These results were validated in a cohort of 12 patients, showing significantly higher levels of miR-92b-3p in synovial sarcoma patients compared with healthy individuals. This miRNA was able to distinguish patients from controls with a sensitivity of 81.1% [69]. Fricke et al. designed a method to detect the fusion transcript in whole blood RNA, RNA from mononuclear cells, and microvesicle RNA, which was tested in a cohort of eight patients and five healthy individuals. The release of microvesicles harboring the *SYT-SSX* fusion by synovial cells was shown in vitro. Nested qPCR, qPCR, nested PCR, and ddPCR were not sensitive enough to detect any fusion transcript in the peripheral blood samples from this small cohort of patients [53].

### 3.2. Myxoid Liposarcoma

Myxoid liposarcoma accounts for 30-50% of liposarcomas, and the majority of the cases are characterized by either t(12;16)(q13;p11) translocation, causing the *FUS-CHOP* product or, more rarely, translocation of t(12;22)(q13;q12), creating the fusion gene *EWSR1-CHOP* [19,54]. These fusion products act as transcription factors and thereby drive tumor progression [19]. In a study by Panagopoulos et al., nested PCR was performed on DNA from circulating tumor cells in the peripheral blood samples of primary and recurrent patients taken prior to surgery. Circulating tumor cells containing this fusion were detected in only four out of 20 samples [54]. The authors suggest the limited sensitivity of the assay to explain the failure of detecting the fusion fragments. Braig et al. designed patient-specific assays to detect *FUS-CHOP* products and *TERT* promotor mutations, which are common in myxoid liposarcomas. In a small cohort of four myxoid liposarcoma patients with active disease, in every patient at least one the aberrations was detected; the quantity of ctDNA correlated with clinical course and disease burden [57].

### 3.3. Alveolar Rhabdomyosarcoma

Rhabdomyosarcoma is the most common soft tissue sarcoma in children and young adults. Rhabdomyosarcoma consists of different entities; in particular, alveolar rhabdomysarcoma has a distinct genetic background including two different translocations. Up to 90% of cases present with either t(2;13) (q35;q14), creating the *PAX3-FOXO1* fusion gene, or t(1;13) (p36;q14), resulting in the *PAX7-FOXO1* fusion product [17,75]. Recently, Eguchi-Ishimae et al. collected a series of cfDNA samples from a patient diagnosed with alveolar rhabdomyosarcoma to examine the fusion sequence *PAX3-FOXO1* as a biomarker. Using nested PCR and qPCR, they were able to detect *PAX3-FOXO1* in ctDNA at relapse and during progression of the disease. In addition, the fusion sequence was detected in plasma ctDNA while the PET-CT had not yet shown the presence of tumor cells, indicating the possibility of ctDNA as a method for the early detection of recurrent disease [75]. A study by Klega et al. used sequencing to detect tumor-specific genomic rearrangements in liquid biopsy samples of pediatric sarcomas, resulting in a detection rate of five out of seven alveolar rhabdomyosarcoma blood samples in a pre-operative setting. For one patient, liquid biopsy samples were collected during chemotherapy treatment and showed a rapid decline in ctDNA levels after the initiation of chemotherapy. At progression, the ctDNA level increased, suggesting a correlation with disease burden and response to therapy [58].

### 3.4. Ewing Sarcoma

Around 85% of Ewing sarcoma cases are driven by the chromosomal translocation t(11;22)(q24;q12), leading to the *EWS-FLI1* fusion protein. The remainder of Ewing sarcomas result from other fusion products such as *EWS-ERG* [76]. As early as 1995, Peter et al. demonstrated a method to detect Ewing sarcoma driving fusion products in peripheral blood and bone marrow samples of 36 Ewing sarcoma patients using RT-PCR followed by nested PCR [55]. Others have found the presence of the fusion sequence to be correlated with tumor burden, thereby suggesting the potential as a biomarker to indicate relapse development [56,59]. More recently, Shulman et al. performed a retrospective analysis to evaluate the association between ctDNA detection and clinical outcome using an NGS method. A total of 94 newly diagnosed or relapsed patients were included in the study; tumor specific fusion sequences were detected in 53.3% of newly diagnosed Ewing sarcomas and 47.1% at relapse. In the group of newly diagnosed patients, ctDNA was detected in 69.2% of patients with metastatic disease compared with 44% of patients with localized disease. When correlating to clinical data, localized Ewing sarcoma with detectable levels of ctDNA had significantly lower event-free survival (EFS) and overall survival (OS) rates, whereas for metastatic Ewing sarcoma with detectable ctDNA, only EFS was shown to be significantly lower [60].

### 3.5. Desmoplastic Small Round Cell Tumor

Desmoplastic small round cell tumor (DSRCT) is a rare, aggressive type of sarcoma characterized by a specific translocation t(11;22)(q13;12) that fuses *EWSR1* to *WT1*. A patient-specific ddPCR was designed after identifying the precise genomic breakpoint of this fusion through sequencing a tumor sample of a patient with a DSRCT. This patient was treated with several forms of systemic therapy and surgery, after which there was no evidence of disease in the imaging. The detection of the fusion sequence as a biomarker was explored to monitor disease during follow-up. ctDNA samples were collected during visits until three years after surgery, and no signs of the fusion sequence were detected in any of these samples. This was in agreement with the favorable clinical response of this patient, showing long-term disease-free survival [77]. The potential of liquid biopsies in DSRCT was also studied by Shukla et al. using two complementary approaches. First, the tumor DNA was sequenced to design a patient-specific ddPCR. Next, a disease-tailored NGS panel was designed to apply to the cfDNA. The small cohort included six DSRCT patients with newly diagnosed, recurrent, or metastatic disease. Tumor specific fusions were successfully identified by ddPCR in five out of six samples, whereas NGS was identified four out of six [61]. In another study by Colletti et al., exosomes from three DSRCT patients and four healthy controls were isolated and analyzed to assess the expression of exosomal miRNA. A panel of 55 miRNAs were significantly differentially expressed in DSRCT patients compared with their matched controls [70]. To explore the clinical utility of ctDNA and miRNA as a marker for treatment response, larger cohorts at different timepoints should be evaluated.

### 3.6. Gastrointestinal Stromal Tumor

ctDNA may be a suitable method to diagnose GISTs based on their tumor-specific mutation status, as mentioned earlier in this review. Besides being a tool for diagnostic purposes, several studies have evaluated the use of ctDNA in GIST for other applications, such as for the prognostication or assessment of tumor heterogeneity. Xu et al. analyzed tumor DNA and matched the plasma ctDNA of 32 advanced GIST patients using an NGS-based multi-gene panel consisting of tumor-related genes, and detected ctDNA mutations in 56.3% of the cases. ctDNA and tissue DNA detection were concordant for 71.9% of the cases. The ctDNA test detected mutations in 18 patients and a normal genotype in 14 patients, whereas the tissue DNA test detected mutations in 25 patients and a normal genotype in seven patients. Concordance was higher for larger tumors and tumors with a higher Ki-67. The number of ctDNA mutations were correlated with tumor size; the positive rate of ctDNA detection was higher in larger tumors (>10 cm) compared with smaller tumors (<10 cm). In addition, ctDNA detection was higher in tumors with Ki-67 detection of >5% compared with tumors with Ki-67 <5%. Tumor size and type of ctDNA mutations were found as independent prognostic factors in this group of patients [62]. Jilg et al. investigated tumor heterogeneity by analyzing the ctDNA of GIST patients. In this study, additional driver mutations were found by applying targeted panel sequencing on cfDNA in addition to PCR in a total of 13 samples of four GIST patients [63]. These additional mutations included aberrations in *TP53*, *NRAS*, *KRAS*, *HRAS*, *PIK3CA*, and *BRAF*. Similar results were obtained in a study by Namløs et al., in which the ctDNA samples showed genomic heterogeneity of the tumor by analyzing different ctDNA samples of one patient [46]. The detection of tumor heterogeneity and the presence of additional driver mutations might indicate treatment resistance in these tumors. In these cases, a treatment switch to an alternative treatment should be considered.

## 4. Liquid Biopsy for Complex Karyotype Sarcomas

In contrast with simple karyotype sarcomas, another subset of sarcomas contains multiple genomic abnormalities for which a liquid biopsy approach by targeting a point mutation or narrow genomic alteration may not be practical. LMS is one of these sarcomas characterized by a highly heterogeneous genomic landscape involving alterations in *TP53*, *RB*, *ATRX*, and *MED12* [78,79]. For this tumor type, Przybyl et al. integrated sequencing protocols to analyze single nucleotide variants (SNVs), insertions or deletions (indels), and CNAs in LMS ctDNA. Seven LMS patients with either a primary tumor or metastatic disease donated serial plasma samples throughout their disease course. Detection of LMS ctDNA based on SNVs and indels was successful in 86% of baseline samples, and demonstrated an overall sensitivity of 68% across all of the samples analyzed. Secondly, CNA analysis was tested for the same purpose and showed an overall sensitivity of 44% across all of the samples. By sequencing the tumor tissue derived from multiple lesions of individual patients, intra-patient variation of mutations was found, indicating the presence of subclones containing different alterations in LMS. ctDNA analysis of CNAs, but not SNVs, demonstrated the detection of these subclonal alterations [64]. Demoret et al. used a commercially available ctDNA panel and compared these results to a tumor comprehensive genomic profiling (CGP) panel to analyze the molecular profiles in both tumor tissue and matched ctDNA samples of 24 patients with advanced STS of different subtypes, including LMS. Of all of the analyzed samples, 75% had detectable ctDNA. Within all of the sarcoma subtypes analyzed, LMS samples showed the best concordance between liquid and solid tumor profiling, and tumor-derived ctDNA was detected for all LMS samples. With these results, the authors suggested LMS as the most potent STS subtype to benefit from liquid biopsy protocols in the future [65]. In a study by Hemming et al., tumor DNA and matched plasma cfDNA samples of 30 LMS patients were evaluated using NGS. In this patient cohort, the tumor burden ranged from no evidence of disease to progressive metastatic disease. The results showed that high levels of ctDNA were associated with an increase in tumor size and disease progression [66].

Osteosarcoma is the most common primary malignant tumor of the bone and is characterized by a complex, heterogeneous karyotype containing numerous genomic alterations as well. Amplifications and loss of heterozygosity are the most frequently found genomic alterations in this type of sarcoma [80]. Thus, detection of osteosarcoma ctDNA requires targeting of multiple commonly mutated genes. Barris et al. studied seven osteosarcoma tumors to identify tumor-specific mutations, and used this for the cfDNA sequencing of tumor matched plasma samples. ctDNA was analyzed at various time points during the disease course and was detected in three out of seven cases, generally during periods of clinical relapse [67]. Shulman et al. developed a method to detect ctDNA without first sequencing the patient’s tumor using banked plasma of 72 osteosarcoma patients with primary localized disease. ctDNA was detected in 57% of samples. In addition, 8q gain was studied among these 41 osteosarcoma patients to investigate its prognostic value, and showed a detection rate of 74.4% among patients with detectable ctDNA [60]. Apart from ctDNA, other techniques have also been studied for the purpose of liquid biopsy or as biomarkers in osteosarcoma, such as various metabolites, microRNAs, and exosomes [68,81].

## 5. Challenges and Perspectives

During the last few years, advances have been made in the area of liquid biopsy for solid cancers for many purposes, including tumor profiling, longitudinal disease monitoring, and for the identification of resistance mechanisms and new targets for therapy. Literature on liquid biopsy in sarcomas remains limited, but the first results are interesting. Clearly, there are still some major issues that need to be addressed to use liquid biopsy in sarcoma clinical practice.

From a technical perspective, sensitivity and specificity remain one of the main issues for most of the analytical assays discussed here, even though there have been general improvements in the sensitivity of methods such as PCR and sequencing. Suboptimal sensitivity increases the risk of false negative results, and, as a consequence, clinicians may miss the presence of disease. The capability to detect the presence of disease using liquid biopsy in sarcoma patients shows a large variation (Table 2). These differences in sensitivity may be caused by different factors, such as the amount of biomarker available, or the type of biomarker and the detection method that is used. Methods to detect CTCs in peripheral blood samples have not shown to be very sensitive in a couple of sarcoma subtypes, including synovial sarcoma, myxoid liposarcoma, and Ewing sarcoma. Even in the metastatic setting, CTCs were not always detected, which may be caused by the low occurrence of CTCs in the blood, the heterogeneity of CTCs, or the lack of a specific marker for detection. For ctDNA approaches, a higher sensitivity was reached by improved PCR protocols, such as ddPCR, showing encouraging results for the detection of Ewing sarcoma, DSRCT, and GIST. These sarcoma subtypes are characterized by specific mutations (GIST) or translocations (DSRCT and Ewing), and are thus well-suited for ddPCR, a method that requires a separate essay set for each specific mutation. Sarcomas with multiple genetic alterations, such as angiosarcoma, osteosarcoma, and leiomyosarcoma, are less amenable for such approaches and require broader targeting. Studies on the more genetically complex sarcomas often use panel sequencing, and although the sensitivity needs to be improved, LMS has been shown as one of the most potent subtypes to benefit from these kind of liquid biopsy protocols in the future [65]. In addition, the determination of tumor fraction for the differentiation between MPNST and PN showed encouraging results, and might improve early cancer detection and monitoring in the future [30]. Furthermore, promising results have been obtained by panel sequencing of ctDNA from GIST patients, which is used to assess mutation status in primary and therapy resistant GIST patients to guide the most optimal therapy choice [43].

Besides technical limitations, the behavior and characteristics of the tumor itself may also influence the ability to detect biomarkers from the circulation. The amount of tumor-derived material shed into the circulation is considered to depend on the tumor histotype and the tumor burden. Although sarcomas are often large masses from which one would expect a high shedding of the tumor material, Serrano et al. found ctDNA shedding to be low in GIST and suggest the same for other mesenchymal tumors [45]. However, less is known about shedding capacities among sarcoma subtypes. In addition, there has been evidence that cfDNA levels fluctuate during the day and show within-subject variation [82]. More studies are warranted to establish these biological variations.

Moreover, evaluating liquid biopsy assays for sarcoma poses a challenge because of the rarity and heterogeneity of the disease. Although universal sarcoma markers for liquid biopsy have been studied [83,84], increasing evidence suggests that assays should be subtype-specific. Even though some studies focusing on a single subtype have shown feasibility, reaching a large sample size to demonstrate predictive value and other pre-analytical factors such as timing of sampling, sample handling, and time to sample processing, remain a main problem. Therefore, multi-center collaborations and sample sharing seem essential to move the field forward. In addition, the rarity of the many different subtypes of sarcomas make it difficult to define the clinical utility of liquid biopsies.

Meanwhile, further studies on sarcoma liquid biopsies are ongoing. As well as the various types of assays described here, there have been other interesting approaches suggested that might be useful in the future. One of the examples is the characterization of ctDNA by the presence of cancer-specific methylation patterns. Several studies have demonstrated the potential of classifying sarcoma subtypes based on the DNA methylation profiles of tumor DNA [85,86]. In a study by Liu et al., investigators found that DNA methylation profiling of cfDNA was able to classify different cancer types [87]. In addition, the use of TEPs as blood-based biomarkers was tested for sarcoma patients recently, and was shown to identify distinct profiles in sarcoma patients compared with controls [88]. Another subject of recent interest is profiling the fragmentation of cfDNA. In a recently published retrospective study of Peneder et al., samples of 95 Ewing sarcoma patients and 31 patients with other pediatric sarcomas were analyzed for their cfDNA fragmentation. This data showed the proportion of short fragments to be higher in cfDNA from patients with Ewing sarcoma compared with the healthy controls [89]. Both methylation and fragmentation could be clinically relevant and need further investigation.

## 6. Conclusions

The discovery of liquid biopsy enables a minimally invasive method for longitudinal disease and therapy monitoring, assessment of tumor heterogeneity, and the identification of resistance mechanisms. Although liquid biopsy has several advantages over traditional biopsy methods, the diagnostic performance varies and is dependent on tumor type. Despite successes in some common cancers, unfortunately, liquid biopsy assays for sarcomas are still in an early phase. The restricted number of available patients and the high heterogeneity between sarcoma subtypes contribute to the limited advances in this field. Although the first results are promising, some sarcoma subtypes such as GIST and LMS may be more suitable for liquid biopsy than others. Concerted effort is needed to evaluate various assays for sensitivity, specificity, and reproducibility in larger, longitudinal trials to show its added value for routine clinical care.

## Figures and Tables

**Table 1:** Methods most frequently used for sarcoma ctDNA analysis. ddPCR: digital droplet PCR; CNA: copy number alteration; NGS: next generation sequencing [20,21,22,23].

Name	Technique	Detection Limit (% ctDNA)	Advantages	Disadvantages
ddPCR	DNA sample is distributed into tiny droplets that are analyzed for the presence of a single mutated or non-mutated DNA strand. The number of positive partitions (in which the sequence is detected) is counted	~0.01%	Rapidly detects specific mutations, low cost, and quantitative	Mutation specific assay, and the number of variants that can be screened is limited
NGS	DNA sample is fragmented into millions of short DNA sequences and analyzed in parallel, followed by either sequence alignment to a reference genome or constructed reference (“de novo sequence assembly”)	~0.01–2%	Capable of screening broader genetic abberations simultaneously, and relative quantitative	Expensive and time consuming, and requires a higher ctDNA input

**Table 2:** Sensitivity and specificity rates of assays performed in sarcoma patients. Rates are either mentioned in the papers or calculated based on provided data. PCR: polymerase chain reaction; ddPCR: digital droplet PCR; RT-PCR: reverse transcription PCR; qPCR: quantitative PCR; NGS: next generation sequencing; L-PCR: ligation PCR; STS: soft tissue sarcoma; DSRCT: desmoplastic small round cell tumor; GIST: gastrointestinal stromal tumor; LMS: leiomyosarcoma; MPNST: malignant peripheral nerve sheath tumor.

Circulating Material	Detection Method	Subtype	Patient Selection	Sensitivity	Specificity	n	References
CTC	Nested PCR and ddPCR	Synovial sarcoma	After primary treatment, various disease stages	0% (nested PCR), 6.7 % (ddPCR)	n/a	15	[51]
	Nested RT-PCR	Synovial sarcoma	Before diagnostic biopsy	5.3%	100%	38 + 18 controls	[52]
	Nested qPCR, qPCR, nested PCR and ddPCR	Synovial sarcoma	Various disease stages, 3 patients on treatment	0%	n/a	13	[53]
	Nested PCR	Myxoid liposarcoma	Various disease stages	n/a	n/a	20	[54]
	RT-PCR	Ewing sarcoma	Various disease stages	n/a	n/a	36	[55]
	RT-PCR	Ewing sarcoma	At diagnosis, localised disease	43%	n/a	7	[56]
	Immunofluorescence	STS (multiple histotypes)	Before/on systemic treatment	n/a	n/a	35	[35]
ctDNA	ddPCR	Myxoid liposarcoma	Various disease stages	n/a	n/a	4	[57]
	NGS	Alveolar rhabdomyosarcoma	Prior to start of different treatments	71.4%	n/a	7	[58]
	ddPCR	Ewing sarcoma	Various disease stages	n/a	n/a	3	[59]
	NGS	Ewing sarcoma	Various disease stages	53% (at diagnosis), 47.1% (relapse)	n/a	94	[60]
	ddPCR	Ewing sarcoma	Various disease stages	n/a	n/a	20	[34]
	ddPCR and NGS	DSRCT	Various disease stages	83% (ddPCR), 67% (NGS)	n/a	6	[61]
	ddPCR	GIST	Various disease stages	92.8% (metastatic), 12.5% (localized)	n/a	22	[28]
	NGS	GIST	Various disease stages	85%	n/a	243	[43]
	NGS	GIST	Advanced disease	n/a	n/a	32	[62]
	NGS	GIST	Various disease stages	n/a	n/a	50	[46]
	ddPCR and NGS	GIST	Various disease stages, *KIT*- or *PDGFRA*- mutant	28.6% (ddPCR), 42.9 % (NGS)	n/a	21	[45]
	L-PCR and ddPCR	GIST	Active disease, *KIT*- or *PDGFRA*-mutant	64% (L-PCR), 80% (ddPCR)	n/a	25	[63]
	NGS	LMS	Metastatic disease	n/a	n/a	73	[42]
	NGS	LMS	Various disease stages	86% (baseline), 44−68% (overall)	98–98.9% (baseline)	7 + 452 controls	[64]
	NGS	LMS	Metastatic disease	100%	n/a	6	[65]
	NGS	LMS	Progressive disease	69%	n/a	16	[66]
	NGS	Osteosarcoma	Various disease stages	50% (active disease), 100% (relapse)	n/a	7	[67]
	NGS	Osteosarcoma	Various disease stages	56.9%	n/a	72	[60]
	NGS and PCR	STS (multiple histotypes)	Non-metastatic disease before and after surgery	n/a	n/a	29	[32]
	NGS	MPNST	During therapy	58%	91%	59 + 14 controls	[30]
	NGS	STS (multiple histotypes)	Metastatic disease	n/a	n/a	11	[33]
miRNA	qRT–PCR	Osteosarcoma	Various disease stages	71.4% (miR-25-3p), 64.3% (miR-17-5p)	92.3% (miR-25-3p), 84.6% (miR-17-5p)	36	[68]
	RT-qPCR	Synovial sarcoma	Various disease stages	81.1% (compared with non-STS patients), 84.6% (compared with other STS subtypes)	63.6% (compared with non-STS patients), 80% (compared with other STS subtypes)	24 +12 controls	[69]
Microvesicles	Nested qPCR, qPCR, nested PCR and ddPCR	Synovial sarcoma	Various disease stages, 3 patients on treatment	0%	n/a	13	[53]
Exosomes	qPCR	DSRCT	Metastatic disease	n/a	n/a	3 + 4 controls	[70]

**Table 3:** Overview of studies of sarcoma liquid biopsy discussed in this review. DSRCT: desmoplastic small round cell tumor; GIST: gastrointestinal stromal tumor; LMS: leiomyosarcoma; MPNST: malignant peripheral nerve sheet tumor; STS: soft tissue sarcoma; SNV: single nucleotide variants; NGS: next generation sequencing; PCR: polymerase chain reaction.

Subtype	Circulating Material	Target	Detection Method	Number of Sarcoma Patients Included	Clinical Implication	References
Synovial sarcoma	CTC	*SYT-SSX* fusion	PCR	1	Prognostication	[73]
	CTC	*SYT-SSX* fusion	PCR	15	Prognostication or surveillance	[51]
	CTC	*SYT-SSX* fusion	PCR	38	Monitoring tumor burden	[52]
	ctDNA	*SYT-SSX* fusion	PCR	1	Monitoring translocation-derived diseases	[74]
	CTC, microvesicles	*SYT-SSX* fusion	PCR	13	Detection of tumor activity	[53]
	miRNA	miR-92b-3p	PCR	21	Monitoring tumor dynamics	[69]
Myxoid liposarcoma	CTC	*FUS-CHOP* fusion, *EWS-CHOP* fusion	PCR	20	Monitoring disease	[54]
	ctDNA	*FUS-CHOP* fusion *TERT* C228T promotor mutation, *FUS-CHOP* fusion	PCR	4	Monitoring disease	[57]
Alveolar rhabdomyosarcoma	ctDNA	*PAX3-FOXO1* fusion	PCR	1	Monitoring tumor burden, and determine diagnosis and treatment options	[75]
	ctDNA	8-gene panel including *EWSR1*, *FUS*, *CIC*, *CCNB3*, *PAX3*, *PAX7*, *STAG2*, *TP53*	NGS	7	Identification of genomic subclassifiers and track disease response	[58]
Ewing sarcoma	CTC	*EWS-FLI1* fusion, *EWS-ERG* fusion	PCR	36	Clinical assessment of dissemination	[55]
	CTC	*EWS-FLI1* fusion	PCR	26	Prediction of recurrent disease and treatment stratification	[56]
	ctDNA	*EWS-FLI1* fusion, *EWS-ERG* fusion	PCR	3	Biomarker of relapse	[59]
	ctDNA	6-gene panel including *EWSR1*, *FUS*, *CIC*, *CCNB3*, *TP53* and *STAG2*,	NGS	94	Prognostication, indicator of chemo responsiveness and minimal residual disease, and treatment stratification	[60]
	ctDNA	*EWS-FLI1* fusion, *EWS-ERG* fusion	PCR	20	Therapy monitoring	[34]
DSRCT	ctDNA	*EWS-WT1* fusion	PCR	1	Disease monitoring	[77]
	ctDNA	3-gene panel including *TP53*, *STAG2* and *CDKN2A*, *EWSR1* fusions	NGS, PCR	6	Diagnostics, prognostication, and monitoring	[61]
	Exosomes	179 miRNA panel	PCR	3	Biomarker to characterize disease status	[70]
GIST	ctDNA	*KIT* exon 11 mutations	PCR	22, 1	Monitoring treatment response	[28,29]
	ctDNA	73-gene panel	NGS	243	Evaluating treatment and managing therapeutic selection	[43]
	ctDNA	22-gene panel, *TP53*	NGS, PCR	1	Therapy monitoring	[44]
	ctDNA	416-gene panel	NGS	32	Diagnostics and prognostication in advanced GIST patients	[62]
	ctDNA	28-ene panel	NGS	50	Capture molecular heterogeneity and guide treatment decisions during progression	[46]
	ctDNA	60-gene panel, *KIT* and *PDGFRA* mutations	NGS, PCR	18	Monitoring tumor dynamics	[45]
	ctDNA	*KIT* and *PDGFRA* mutations	PCR	25	Indicator of disease activity and companion biomarker	[63]
LMS	miRNA	miR-25-3p	miRNA array	6	Prediction of diagnosis	[31]
	ctDNA	73-gene panel	NGS	73	Identification genomic alterations and development of targeted therapies	[43]
	ctDNA	89-gene panel	NGS	7	Disease monitoring	[64]
	ctDNA	62-gene panel	NGS	6	Disease monitoring	[65]
	ctDNA	Genome wide	NGS	30	Guiding treatment decisions, monitoring response, surveying for disease recurrence, and differentiating benign and malignant tumors	[66]
Osteosarcoma	ctDNA	7-gene panel including *MET*, *PTEN*, *DLG2*, *RB1*, *TP53*, *SLC19A1*, *ATRX*	NGS	7	Monitoring clinical outcomes and investigate actionable targets	[67]
	ctDNA	Genome wide, focused on 8q gain	NGS	72	Prognostication, indicator of chemo responsiveness, and marker of minimal residual disease	[60]
	miRNA	miR-25-3p	miRNA array, PCR	10	Tumor monitoring and prognostic prediction	[68]
MPNST	ctDNA	Genome wide copy number alterations, focused on *NF1*, *SUZ12*, *SMARCA2*, *CDKN2B*, *CDKN2A*, 8q, 9q, *EED*, *MDM2*, *TP53*	NGS	14	Early detection, treatment response	[30]
Liposarcoma	ctDNA	*TP53*	NGS	17	Therapy monitoring	[47]
STS (multiple histotypes)	ctDNA	3-gene panel including *RB1*, *TP53*, *ATRX*, 12 genes for ddPCR, 30 SNVs for intra operative plasma samples	NGS, PCR	29	Disease monitoring	[32]
	ctDNA	13-gene panel	NGS	11	Characterize ctDNA in metastatic sarcoma	[33]
	CTC	Number of CTCs	Immunofluorescence	35	Disease monitoring	[35]
